# One Size Fits All? Standardised Provision of Care for Survivors of Sexual Violence in Conflict and Post-Conflict Areas in the Democratic Republic of Congo

**DOI:** 10.1371/journal.pone.0111096

**Published:** 2014-10-20

**Authors:** Jerlie Loko Roka, Rafael Van den Bergh, Sokhieng Au, Eva De Plecker, Rony Zachariah, Marcel Manzi, Vincent Lambert, Elias Abi-Aad, Kassi Nanan-N’Zeth, Serge Nzuya, Brigitte Omba, Charly Shako, Derick MuishaBaroki, Jean Paul Basimuoneye, Didier Amudiandroy Moke, Emmanuel Lampaert, Lucien Masangu, Anja De Weggheleire

**Affiliations:** 1 Médecins Sans Frontières, Operational Centre Brussels, DRC Mission, Kinshasa, Democratic Republic of Congo; 2 Médecins Sans Frontières, Operational Centre Brussels, Operational Research Unit (LuxOR), Luxembourg, Luxembourg; 3 Médecins Sans Frontières, Operational Centre Brussels, Medical Department, Brussels, Belgium; 4 Médecins Sans Frontières, Operational Centre Brussels, Operations Department, Brussels, Belgium; 5 Ministère de la Santé, Bureau Central Zone de Santé Masisi, Masisi, Democratic Republic of Congo; 6 Ministère de la Santé, Bureau Central Zone de Santé Niangara, Niangara, Democratic Republic of Congo; University of Oxford, Kenya

## Abstract

**Background:**

Outcomes of sexual violence care programmes may vary according to the profile of survivors, type of violence suffered, and local context. Analysis of existing sexual violence care services could lead to their better adaptation to the local contexts. We therefore set out to compare the Médecins Sans Frontières sexual violence programmes in the Democratic Republic of Congo (DRC) in a zone of conflict (Masisi, North Kivu) and post-conflict (Niangara, Haut-Uélé).

**Methods:**

A retrospective descriptive cohort study, using routine programmatic data from the MSF sexual violence programmes in Masisi and Niangara, DRC, for 2012.

**Results:**

In Masisi, 491 survivors of sexual violence presented for care, compared to 180 in Niangara. Niangara saw predominantly sexual violence perpetrated by civilians who were known to the victim (48%) and directed against children and adolescents (median age 15 (IQR 13–17)), while sexual violence in Masisi was more directed towards adults (median age 26 (IQR 20–35)), and was characterised by marked brutality, with higher levels of gang rape, weapon use, and associated violence; perpetrated by the military (51%). Only 60% of the patients in Masisi and 32% of those in Niangara arrived for a consultation within the critical timeframe of 72 hours, when prophylaxis for HIV and sexually transmitted infections is most effective. Survivors were predominantly referred through community programmes. Treatment at first contact was typically efficient, with high (>95%) coverage rates of prophylaxes. However, follow-up was poor, with only 49% of all patients in Masisi and 61% in Niangara returning for follow-up, and consequently low rates of treatment and/or vaccination completion.

**Conclusion:**

This study has identified a number of weak and strong points in the sexual violence programmes of differing contexts, indicating gaps which need to be addressed, and strengths of both programmes that may contribute to future models of context-specific sexual violence programmes.

## Introduction

Sexual violence is a devastating problem worldwide, having a major impact on the medical and psychological wellbeing of its survivors [1]. In particular in unstable areas such as conflict and post-conflict zones, certain (sub-)populations are highly vulnerable to sexual violence. In an active conflict, militias are frequently perpetrators of (often brutal) sexual violence, using it as a weapon of war against the local population [2], [3]. In post-conflict zones, social destabilization due to population displacements and past traumatic experiences tied to conflicts can lead to high levels of abuse within families and communities [3]. For survivors, sexual violence can lead to mental and physical problems, and psychological post-traumatic reactions or disorders such as anxiety or depressive disorders, suicide attempts, and substance abuse. Sexual violence against children in particular destroys societal structures and can gravely destabilize communities.

In settings such as the Democratic Republic of Congo (DRC), where security is compromised due to frequent conflicts, and women in particular are left vulnerable due to lower societal status, sexual violence is highly prevalent. An estimated 1.7 to 1.8 million women have suffered from sexual violence in the DRC, with more than 400,000 new cases each year [4]. Available data on male survivors of sexual violence are much more rare [5]. For many years now, sexual violence has been widespread in the eastern DRC, but with the recent further deterioration of the security situation, the level and brutality of sexual violence has reached epidemic proportions [6]. A recent study suggests that for eastern DRC alone, approximately 1.3 million women and 760,000 men have fallen victim to sexual violence [7].

Even though the DRC has been termed the “rape capital” of the world [8], and the extraordinary brutality (including gang rape, sexual slavery, and mutilation) in the conflict-afflicted eastern DRC has been reported frequently by different organisations in the region, the international community has failed to respond in an organised or adequate manner. Humanitarian organisations such as Médecins Sans Frontières (MSF) offer care to survivors of sexual violence when possible, but programmes for the care of such survivors rarely undergo formal evaluation, and few models of comprehensive care exist [9]. Depending on the profile of the survivors, the type of sexual violence suffered, and the local context, programme performance may be variable. Documentation and analysis of existing services for sexual violence survivors could lead to better adaptation of such programmes to the survivors’ needs and to the local contexts. With these goals in mind, the objectives of this study are to compare and contrast the patterns of sexual violence, the characteristics of its survivors, and the components of its management in the MSF sexual violence programmes in a context of conflict (Masisi in North Kivu province) and in the post-conflict region of Niangara (Haut-Uélé in Orientale province).

## Methods

### Study design

A retrospective cohort study utilizing routine programmatic data from MSF programmes for sexual violence survivors in Masisi and Niangara, DRC.

### Study setting and population

The study is set in the DRC, one of the countries with some of the worst health and socio-economic indicators globally [10], and having a long history of unrest and instability. Data were collected from January to December 2012 in two MSF programmes offering care to sexual violence survivors: one in an active conflict zone in the remote Masisi Health Zone in North Kivu province (north-eastern DRC), and one in the remote, post-conflict Niangara Health Zone in Orientale province in northern DRC.

Masisi is a zone of open conflict between the national forces of the DRC (*Forces Armées de la République Démocratique du Congo* (FARDC)) and various armed militias, with high reported rates of sexual violence against both women and men committed by military and paramilitary groups. Niangara, in contrast, is a post-conflict region, where the population has suffered for many years from depredations from armed rebel forces, with a fragile peace only recently being established. In both settings, MSF collaborates with the Ministry of Health *(Ministère de la Santé Publique)* in projects offering free access to general health care, consisting of a comprehensive support package to the referral hospital and outlying primary health care clinics (two of which with direct MSF support, in both Masisi and Niangara), an ambulance service and – in the case of Masisi – a network of mobile clinics. Sexual violence care (see below) is managed as an integrated component in both projects, following national and MSF guidelines. These are in accordance with the WHO guidelines on medical management of sexual violence survivors: only post-exposure prophylaxis (PEP) as done by MSF is more up-to-date than the current WHO protocols [11]–[13].

### Sexual Violence Programmes of MSF

Sexual violence is defined as “any sexual act, attempt to obtain a sexual act, unwanted sexual comments or advances, or acts to traffic, or otherwise directed, against a person’s sexuality using coercion, by any person regardless of their relationship to the victim, in any setting, including but not limited to home and work” [1]. Sexual violence includes rape, attempted rape, collective rape and other forms of aggression involving a sexual organ.

Awareness on sexual violence care was raised principally through community meetings and, in the Masisi programme, through the *mamans conseillères* (“counsellor mothers”), who were community reference persons based in strategic locations in the area. Since 2008, eight networks of a total of 66 *mamans conseillères* were set up: such individuals received a training on sexual violence (including which services were offered, and why and when survivors should seek care), and were tasked which conducting health talks on sexual violence in their village, motivating survivors to seek care, explaining that confidentiality was assured, and providing referral to health centres upon request. The *mamans conseillères* received in-kind incentives, for an amount of 15 USD per month. In order to improve the follow-up and management of the *mamans conseillères*, numbers were reduced to approximately 40 after July 2011, when a sufficient awareness among the population was deemed to have been reached, and further to approximately 20 over the course of 2012.

In Niangara, sexual violence awareness was raised through a drama/theatre performance conducted by health promoters in the villages in the region and in the hospital. These performances focused on sexual violence only, and had three main themes: access to care and the consequences of not presenting for care; the practice of settlements out of court with known perpetrators, often leading to the survivor not seeking care; and feelings of guilt among survivors, in particular when they are accused of consenting to the sexual acts. Performances were accompanied by health promotion messages (including the type of care provided, the free and confidential nature of care, the importance of presenting within 72 hours, the type of cases taken in charge (i.e. only sexual violence), and the persons to address at the health structure when presenting after sexual violence), and were followed by a public discussion and question & answers session on the scenario. Performances were usually well-attended and well-received.

Comprehensive medical and psychological care for survivors was provided at the general hospitals of Masisi and Niangara: survivors presented either directly to the hospital, or were referred through district health centres: in Masisi, five health centres (Bihambwe, Buguri, Kanyatsi, Mahanga and Nyabiondo) provided first line medical care (such as HIV post-exposure prophylaxis (PEP) and emergency contraception, but not vaccination, due to the lack of cold chain), while in Niangara two health centres (Tapili and Nambia) offered first-line medical care for survivors and referred to the hospital only for psychological care. In both settings, care was provided by specifically trained and designated staff – first-line medical care was provided by nurses, with back-up support of a doctor at the hospitals. A full package of care was offered in a designated room, this to offer a one-stop strategy to maximise comfort and confidentiality of the patient. In this way, excessive waiting times for patients were avoided by performing registrations with dedicated staff and offering integrated pharmacy care in the designated room rather than through the central pharmacy, and confidentiality was protected by avoiding communal waiting rooms.

The comprehensive package of care offered by MSF to sexual violence survivors included a full medical examination (including a genital and/or anal examination, opt-out offer of HIV counselling and testing, and pregnancy testing), medical care (emergency contraception (for all females aged 12–45, presenting within 120 hours after rape), prophylaxis for sexually transmitted infections (STI – for all rape survivors), HIV PEP (for all rape survivors presenting within 72 hours), vaccination for hepatitis B and tetanus, and wound care if indicated), psychological counselling, preparation of a medico-legal certificate, medico-legal support if requested, and safe shelter and external referral for social assistance for specific cases in Masisi. Systematic follow-up appointments were made for psychological care and for specific circumstances such as pregnancy. All care was provided free of charge.

### Data and Statistical Analysis

All data of sexual violence survivors were entered into a dedicated Excel database and were analysed using the EpiData Analysis v2.2.1.173 software package (EpiData Association, Odense, Denmark). Simple summary statistics are presented, and differences in proportions between conflict and post-conflict settings were assessed by chi-square test. Additionally, a spatial mapping of reported sexual violence in Masisi was performed to assess the frequency and distribution of incidents.

### Ethics considerations

The study has satisfied the Médecins Sans Frontières Ethics Review Board (Geneva, Switzerland) criteria for studies using routinely collected data, and was approved by the Comité d’Ethique de l’Ecole de Santé Publique de l’Université de Kinshasa, DRC (ESP/CE/089/13). The study was conducted as retrospective analysis of routine programme data, and informed consent was thus not sought from study subjects; however, identifying information was removed from all patient records prior to analysis.

## Results

### Survivor demographics

**Figure 1:**
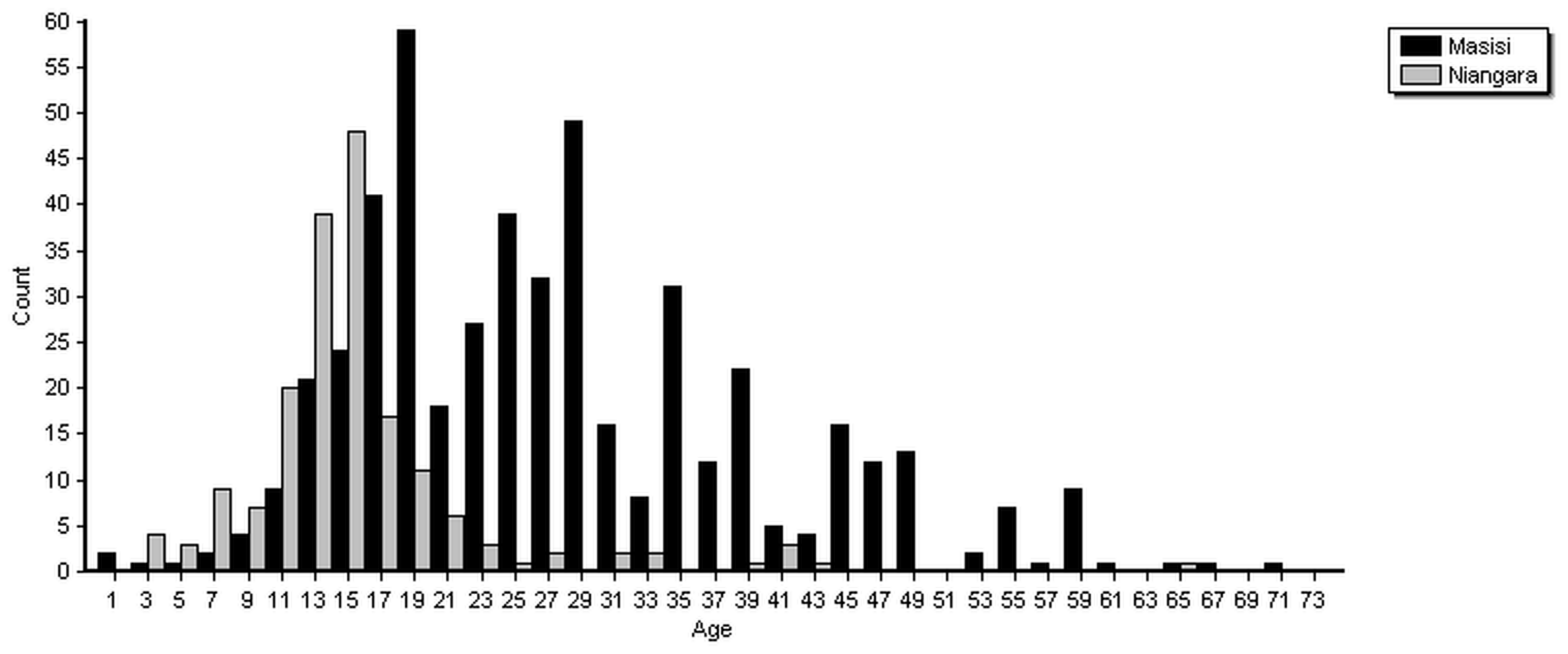
Age histogram of survivors of sexual violence attending the Médecins Sans Frontières sexual violence care programmes in Masisi (conflict zone) and Niangara (post-conflict zone), Democratic Republic of Congo, 2012.

In Masisi, 491 survivors of sexual violence presented for care, compared to 180 in Niangara. Differences in age distribution were observed, with survivors in Masisi being older of age, while those in Niangara were predominantly children and adolescents (fig. 1). The median age in years at the time of assault was 26 (interquartile range (IQR) 20–35) in Masisi and 15 (IQR 13–17) in Niangara. In both settings, women presented the vast majority of survivors, with only 8 men (2%) in Masisi and 1 (1%) in Niangara presenting for care.

### Presentation for care

Characteristics of referral and presentation for care are provided in table 1∶60% of the patients in Masisi arrived for a consultation within the critical time frame of 72 hours, when HIV PEP and prophylaxis for STI’s is most effective, and cumulatively 63% within five days, when emergency contraceptives can be effectively given. In Niangara, only 32% and 42% arrived within these respective timeframes (table 1). The principal reason given for the delay in Niangara was fear (22% of patients) while the predominant reason in Masisi was lack of knowledge on the available treatment (29%). In Masisi, most survivors were self-referred or referred through a friend (40%), while in Niangara, most survivors were referred through another NGO (30%). In Masisi, the community activities (including the *mamans conseillères* network) was responsible for linking 20% of the patients to care (compared to 11% in Niangara), while the theatre/drama approach (inexistent in Masisi) in Niangara managed to link 17% of the patients to care.

**Table 1:** Characteristics of presentation to sexual violence programmes in Masisi (conflict zone) and Niangara (post-conflict zone), Democratic Republic of Congo, 2012.

	Masisi		Niangara		
	N	%	N	%	p-value*
**Total**	**491**		**180**		
**Referral**					**<0.0001**
Self or relative/friend	195	40%	47	26%	
Community talks/community member**	97	20%	20	11%	
Theatre awareness campaign	0	0	31	17%	
Other NGO	75	15%	54	30%	
Medical structure	71	15%	17	9%	
Police	11	2%	8	4%	
Other	42	9%	3	2%	
**Time passed before presenting for treatment**					**<0.0001**
<72 h	295	60%	58	32%	
72 −120 h	15	3%	18	10%	
6 days −1 month	65	13%	46	26%	
1 month −1 year	75	15%	46	26%	
>1 year	41	8%	12	7%	
**Cause of delay in consultation**	**196**		**122**		**<0.0001**
No access to health structures	32	16%	11	9%	
Lack of knowledge of treatments	57	29%	14	12%	
Fear	29	15%	27	22%	
Shame	13	7%	0	0	
Other	65	33%	67	57%	
Not entered	0	0	3	3%	

*Chi-square test for proportions; **: including *mamans conseillères* in the Masisi programme; NGO: non-governmental organisation.

**Figure 2:**
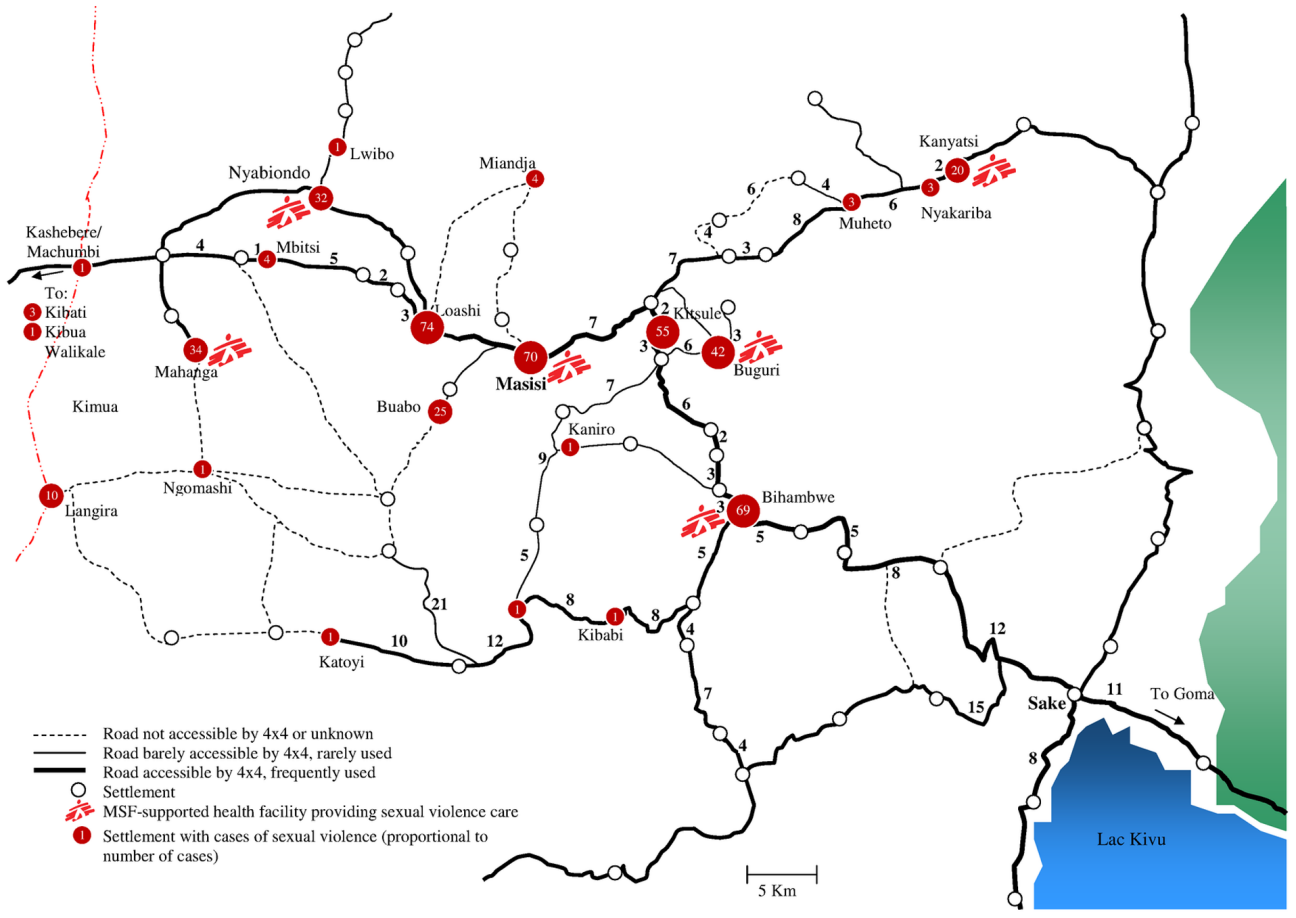
Distribution of sexual violence cases attending the Médecins Sans Frontières sexual violence care programme in Masisi, Democratic Republic of Congo, 2012.

In the Masisi context, where mobility was poor and populations were exposed to the localized presence of armed military groups, the geographical distribution of sexual violence cases was mapped, revealing that survivors mainly came from within a radius of 30 kilometers around the hospital (fig. 2). Most cases (55%) came from zones with direct MSF support (Masisi and the five health areas where MSF directly supports the health centres for sexual violence care), or from the health areas of Loashi (15%) and Kitshulé (11%).

### Characteristics of sexual violence

Rape was the predominant form of sexual violence reported in both projects (table 2). In Niangara, known civilians were the main perpetrators (48%), while in Masisi, the military were the principal aggressors (51%). In general, sexual violence in Masisi was characterized by more brutal forms of assault, with higher levels of gang rape, weapon use, and associated violence. Physical trauma, however, was more frequent in Niangara, where 69 cases (38%) showed a form of trauma – out of these, 50 (72%) were cases presenting with hymen tears, likely reflecting the young age of the survivors.

**Table 2:** Characteristics of sexual violence among survivors presenting for care in Masisi (conflict zone) and Niangara (post-conflict zone), Democratic Republic of Congo, 2012.

	Masisi		Niangara		
	N	%	N	%	p-value*
**Type of aggression**					0.3**
Rape	477	97%	171	95%	
Sexual Touching	5	1%	3	2%	
Forced to rape	6	1%	1	1%	
Non-sexual aggression	3	1%	5	3%	
**Number of perpetrators**					0.06**
1	363	74%	150	83%	
2–4	110	22%	30	17%	
5 and more	15	3%	0	0	
Unknown	3	1%	0	0	
**Profile of perpetrator**					**<0.0001**
Known civilian	116	24%	86	48%	
Unknown civilian	71	15%	42	23%	
Military	251	51%	19	11%	
Family member	0	0	31	17%	
Other	53	11%	2	1%	
**Weapon use**					**<0.0001**
Armed assault	321	65%	8	5%	
Associated violence***					**<0.0001**
Any associated violence	176	36%	34	19%	
**Physical trauma**					**<0.0001**
Any physical trauma	51	10%	69	38%	

*Chi-square test for proportions, except where indicated; **Chi-square test with Yates’ correction; *** Including beatings, mutilations, robbery, destruction of property, rape in public and sexual exploitation.

### Management of sexual violence survivors

Characteristics of patient management are indicated in table 3. Psychological counselling was provided at all intake and follow-up consultations. In general, follow-up of patients was challenging, with only 49% of all patients in Masisi and 61% in Niangara returning for at least one follow-up consultation, and only 4% and 12% in the respective areas returning for their fourth follow-up consultation (a total of five visits is needed to complete the vaccination schedule, and four visits are strongly recommended in the WHO guidelines [11], [12]). Consequently, coverage of most components of sexual violence care given at first visit (PEP initiation, STI prophylaxis, emergency contraception, pregnancy testing) was relatively high, except for vaccination and HIV testing, which were only conducted systematically in the central hospitals and were rarely/not done in the MSF-supported health centres (data not shown). Coverage of follow-up activities (HIV testing after postponing, completion of PEP, completion of vaccination), on the other hand, was low overall.

**Table 3:** Treatment characteristics of sexual violence survivors presenting for care in Masisi (conflict zone) and Niangara (post-conflict zone), Democratic Republic of Congo, 2012.

	Masisi		Niangara		
	N	%	N	%	p-value*
Follow-up**					**<0.0001**
First return visit	239/491	49%	109/180	61%	
Second return visit	137/491	28%	68/178	38%	
Third return visit	51/462	11%	35/155	23%	
Fourth return visit	15/354	4%	13/109	12%	
**HIV testing at first contact**					<0.0001***
Turned down	7	1%	5	3%	
Test not available	292	60%	44	25%	
Status already known	1	0.2%	2	1%	
Postponed to a later date	9	2%	106	59%	
Consented to testing	181	37%	21	12%	
Not documented	1	0.2%	2	1%	
**HIV testing at any visit**	**211**	**43%**	**38**	**21%**	0.7***
Positive	4	2%	1	3%	
Negative	207	98%	37	97%	
**PEP Prophylaxis**					
Eligible (all survivors of rape presenting within 72 h)	288	59%	51	29%	<0.0001
Started (out of eligible)	284	99%	51	100%	0.4
Completed (out of started)	56	20%	20	39%	0.002
**STI Prophylaxis**					
Eligible (all survivors of rape)	483	98%	172	96%	0.03
Started (out of eligible)	480	99%	164	95%	0.0004
**Emergency Contraception**					
Eligible (all female survivors of rape age 12–45 presenting within 5 days)	274	56%	53	29%	<0.0001
Started (out of eligible)	250	91%	46	87%	0.3
**Pregnancy test**					
Eligible (all female patients age 12–45)	424	86%	148	82%	0.2
Test done (out of eligible)	408	96%	130	88%	0.0002
Test positive (out of done)	18	4%	19	15%	0.0001
Abortion requested (out of pregnant)	2/18	17%	12	63%	0.01
Abortion done (out of requested)	0	0	9	75%	0.05
**Tetanus vaccination**					
TT1	334	68%	138	77%	0.03
TT2 (out of TT1)	59	18%	36	26%	0.04
**Hepatatis B vaccination**					
HepB1	266	54%	142	79%	<0.0001
HepB2 (out of HepB1)	161	61%	83	59%	0.7

*Chi-square test for proportions; **Follow-up completion was assessed at 7 days (visit 1), 28 days (visit 2), 3 months (visit 3), and 6 months (visit 4); denominators are the number of patients who were eligible for these follow-up visits; ***Chi-square test with Yates’ correction.

PEP: Post-Exposure Prophylaxis; STI: Sexually Transmitted Infection.

## Discussion

This is among the first studies to compare sexual violence programmes in conflict and post-conflict settings. It identified marked differences in sexual violence and uptake of care between the two contexts, such as higher levels of brutality in Masisi, and higher rates of child abuse and longer delays before presentation for care in Niangara. It also highlights similarities in uptake of these programmes in the two settings: low numbers of patients returning for follow-up care, and challenges in drawing in patients during the crucial 72-hour window period after the sexual violence incident.

According to the WHO report on sexual violence [1], young women are at greater risk of rape than older women, an observation which was also made in Nigeria [14]. Our findings confirm this notion in post-conflict zones, but indicate a different tendency in conflict zones, where women of all ages were seen in our programmes. Similar results were reported in this region in other programmes [15]. Sexual violence constitutes a weapon of war in the DRC and its severity and ubiquity is such that the eastern DRC is considered to be one of the worst places in the world to be female, regardless of age [8]. Male survivors were rarely encountered in the programmes, despite published evidence that sexual violence also frequently targets men in these settings, suggesting a shortcoming of the programme in this regard [5].

Our results indicated that more than 50% of the aggressors in the conflict zone were military, similar to other studies in these contexts [2]; however, it is interesting to note that known or unknown civilians were still responsible for a substantial proportion of aggressions. In the post-conflict context, civilians known to the survivor were the most common perpetrators. Similar observations have been made for both the DRC and other post-conflict regions in West Africa, and reveal the multifactorial causes of sexual aggression [16], [17]. This represents a considerable challenge to an organisation such as MSF, which has a focus on medical curative care and humanitarian relief and does not have the capacity to work on long-term prevention activities in collaboration with local actors. Better collaboration and partnerships with local and international NGO’s and community groups are thus needed to cover this gap.

Achieving adequate vaccination coverage seemed the most problematic aspect of medical care, mainly related to challenges concerning the cold chain in the more remote health centres providing sexual violence care – for this reason, it was not included as part of the health centre package of care. For tetanus vaccination, an additional factor could be that it is included in the Extended Programme of Immunization (EPI), and some women were likely vaccinated through this system.

Unquestionably, abortion constitutes a crucial and acute problem in DRC, a country which forbids all voluntary abortion [18]. The occurrence of a pregnancy due to sexual violence constitutes an additional factor of distress for the survivors. Even though the proportion of pregnancies arising from sexual violence is low, they do occur, and survivors seeking a termination through the public health system are often met with the response that “nothing can be done.” The decriminalization of abortion or, at minimum, a relaxation of the law for survivors of sexual violence would eliminate this double burden.

The care offered by MSF in the two health zones was intended to be comprehensive for medical and psychological care. Data on psychological care of survivors was recorded in the independent database of the general mental health component of the programme, and cross-linking of psychological and medical data of survivors was not possible, representing an important study weakness. Integration of both databases would be an important step forward for the monitoring and analysis of the integrated care offered in our programmes. Additionally, for future evaluations of sexual violence programmes we recommend qualitative approaches to complement the current quantitative analysis, which would allow the vision and the experiences of the involved communities on the sexual violence programmes to be taken into account.

The medical care provided followed the recommendations of the WHO [11], [12]. The efficacy of medical care is highly dependent on the delay in seeking medical care, and HIV PEP and STI prophylaxis are most effective within 72 hours of exposure. Only 60% of the survivors consulted within this critical time period in Masisi, while this was only 32% in the Niangara health zone, with as direct consequence a low initiation of HIV PEP. These results, supported by similar observations elsewhere [9], revealed the need for improved awareness-raising and education among the public, as a major part of the delay in seeking treatment is still a lack of information [19] – in Niangara, the practice of out-of-court settlements between the family of the survivor and the lack of knowledge that this should not preclude seeking medical care further compound this problem. In Masisi, the strategy of MSF for public health education on sexual violence was essentially based on the *mamans conseillères*, living in the community and charged with delivering these messages and guiding survivors towards appropriate structures in a confidential manner. This strategy promotes contact and quickly establishes a trust between the “counsellor” and the survivor, with an added advantage that it limits the number of intermediaries interviewing the survivor before presentation for medical care and maintains confidentiality, but it is limited in the number of people it can reach directly. In this regard, the theatre/drama approach implemented in Niangara may be a promising strategy on awareness raising and first contact with survivors. Additionally, as the mapping of sexual violence cases in Masisi indicated, most survivors who present for care lived close to an MSF-supported facility – decentralising services further by involving more health centres as first-line sexual violence care centre could improve timely access to care. Technical provisions should be made at such health centres to ensure full coverage of first-line medical care, such as vaccination (currently not included in the package of care) and HIV testing. The poor availability of HIV tests in the MSF-supported health centres in Masisi, leading to 60% of the cases remaining untested at first contact, illustrates this challenge, and may lead to reduced trust of survivors in the services offered. The packaging of HIV tests in particular is worth addressing, as it is currently not convenient for use at health centre level (packaged at ≥20 tests/pack, and short shelf life). Overall, our results carry implications for the current strategy of providing standardised sexual violence care. The differences between the characteristics of sexual violence and sexual violence care in the two settings illustrate how programmes can benefit from a good knowledge of the profile of their beneficiaries, and should be tailored to the local context: programmes in post-conflict areas could focus on care of younger survivors, and protection against abuse by family members/known civilians, while programmes in conflict areas need to be prepared for more brutal forms of sexual violence, with increased needs for (decentralised) medical and psychological care. Post-conflict areas need to develop mechanisms of reducing the fear of repercussions from the known perpetrators and of addressing the apparent impunity of such perpetrators, while conflict areas may need more awareness raising in general. One standard of care for all sexual violence programmes, regardless of the context, may therefore not be ideal.

In conclusion, this study has identified a number of weak and strong points in the sexual violence programmes of the differing contexts of Masisi and Niangara, indicating gaps which need to be addressed, and strengths of both programmes, which may contribute to models of context-specific sexual violence programmes in the future.
